# Evaluating machine learning algorithms to Predict 30-day Unplanned REadmission (PURE) in Urology patients

**DOI:** 10.1186/s12911-023-02200-9

**Published:** 2023-06-13

**Authors:** Koen Welvaars, Michel P. J. van den Bekerom, Job N. Doornberg, Ernst P. van Haarst, OLVG Urology Consortium, J. A. van der Zee, G. A. van Andel, B. W. Lagerveld, M. C. Hovius, P. C. Kauer, L. M. S. Boevé

**Affiliations:** 1 Data Science Team, OLVG, Jan Tooropstraat 164, 1061 AE Amsterdam, the Netherlands; 2 Department of Orthopaedic Surgery, UMCG, Groningen, Netherlands; 3 Department of Orthopaedic Surgery, OLVG, Amsterdam, Netherlands; 4 Faculty of Behavioural and Movement Sciences, Department of Human Movement Sciences, Vrije Universiteit Amsterdam, Amsterdam Movement Sciences, Amsterdam, the Netherlands; 5 Department of Urology, OLVG, Amsterdam, the Netherlands

**Keywords:** Unplanned readmissions, Urology, Machine Learning, Algorithms, XGBoost

## Abstract

**Background:**

Unplanned hospital readmissions are serious medical adverse events, stressful to patients, and expensive for hospitals. This study aims to develop a probability calculator to **p**redict **u**nplanned **re**admissions (PURE) within 30-days after discharge from the department of Urology, and evaluate the respective diagnostic performance characteristics of the PURE probability calculator developed with machine learning (ML) algorithms comparing regression *versus* classification algorithms.

**Methods:**

Eight ML models (i.e. logistic regression, LASSO regression, RIDGE regression, decision tree, bagged trees, boosted trees, XGBoost trees, RandomForest) were trained on 5.323 unique patients with 52 different features, and evaluated on diagnostic performance of PURE within 30 days of discharge from the department of Urology.

**Results:**

Our main findings were that performances from classification to regression algorithms had good AUC scores (0.62–0.82), and classification algorithms showed a stronger overall performance as compared to models trained with regression algorithms. Tuning the best model, XGBoost, resulted in an accuracy of 0.83, sensitivity of 0.86, specificity of 0.57, AUC of 0.81, PPV of 0.95, and a NPV of 0.31.

**Conclusions:**

Classification models showed stronger performance than regression models with reliable prediction for patients with high probability of readmission, and should be considered as first choice. The tuned XGBoost model shows performance that indicates safe clinical appliance for discharge management in order to prevent an unplanned readmission at the department of Urology.

## Plain Language Summary

**Study need and importance:**

Unplanned readmissions form a consistent problem for many hospitals. Unplanned readmission rates can go up as high as to 35%, and may differ significantly between respective hospital departments. In addition, in the field of Urology readmission rates can be greatly influenced by type of surgery performed and unplanned readmissions in patients can go up as high as 26%. Although predicting unplanned readmissions for individual patients is often complex, due to multiple factors that need to be taken into account (e.g. functional disability, poor overall condition), there is evidence that these can be prevented when discharge management is evaluated with an objective measuring tool that facilitate such risk stratification between high and low risk patients. However, to the best of our knowledge, the latter risk stratification using ML driven probability calculators in the field of Urology have not been evaluated to date. Using ML, calculated risk scores based on analysing complex data patterns on patient level can support safe discharge and inform concerning the risk of having an unplanned readmission.

**What we found:**

Eight ML models were trained on 5.323 unique patients with 52 different features, and evaluated on diagnostic performance. Classification models showed stronger performance than regression models with reliable prediction for patients with high probability of readmission, and should be considered as first choice. The tuned XGBoost model shows performance that indicates safe clinical appliance for discharge management in order to prevent an unplanned readmission at the department of Urology. Limitations of our study were the quality and presence of patient data on features, and how to implement these findings in clinical setting to transition from predicting to preventing unplanned readmissions.

**Interpretation for clinicians:**

ML models based on classification should be first choice to predict unplanned readmissions, and the XGBoost model showed the strongest results.

## Introduction

Unplanned readmissions form a consistent problem for many hospitals, rates can go up as high as to 35%, and differ significantly between hospital departments [1]. Departments with a heterogenous patient population often experience high unplanned readmission rates (e.g. Intensive Care Unit (ICU), Internal medicine, Geriatric medicine) due to the complexity of care, heterogenous patient population, and suboptimal discharge management on individual patient level [2]. In addition, in the field of Urology readmission rates can be greatly influenced by type of surgery performed and readmissions in patients can go up as high as 26% [3]. Although predicting unplanned readmissions for individual patients is often complex, due to multiple features that need to be taken into account (e.g. functional disability, poor overall condition), there is evidence that these can be prevented when discharge management is evaluated with an objective measuring tool that facilitate such risk stratification between high and low risk patients [4, 5]. The latter risk stratification using Machine Learning (ML) driven probability calculators in the field of Urology have not been evaluated to date.

Using ML, calculated risk scores based on analysing complex data patterns can support safe discharge on patient level, and can be used with capacity management on a department level. The physician team can assess high-risk scores by evaluation of the responsible modifiable (i.e. can act on) risk factors on patient level. With this information, the physician team may evaluate if the patient is safe for discharge, needs to stay admitted in order to optimize specific modifiable features, and if discharged whether bed capacity needs to be taken into account for possible unplanned readmission. The use of such ML driven algorithms in clinical setting has shown to be feasible application in predicting unplanned readmissions [6]. Moreover, shared decision-making based on individualised risk stratification reduces the risk of unplanned readmission up to 13%. This includes informing the patient about the current situation, optimizing specific features before discharge, and discussing what factors (i.e. features) carry risk and could lead to an unplanned readmission [7].

From a ML methodological point of view algorithms are commonly trained with limited set of features (i.e. variables), such as length of stay, acuity of admission, comorbidity, and emergency department utilization in the 6 months before admission (LACE). While larger sets of features are available in the patient chart during clinical admission which can be applied to train algorithms with [8, 9]. Also, there are few comparisons between regression and classification based algorithms in context of unplanned readmissions [10].

Our primary aim was to develop a ML-driven probability calculator to **p**redict **u**nplanned **re**admissions (PURE) within 30-days after discharge for patients that had a clinical admission at the department of Urology. Our second aim was to evaluate the difference performance of the PURE probability calculator developed using ML algorithms, comparing regression *versus* classification algorithms. We hypothesized it is feasible to develop a strong performing PURE probability calculator, and there is no difference in performance when developed with ML algorithms using classification *versus* regression algorithms.

## Methods

### Guidelines

This study followed the guidelines for Developing and Reporting Machine Learning Predictive Models in Biomedical Research, and the guidelines for Transparent Reporting of Multivariable Prediction Models for Individual Prognosis or Diagnosis (TRIPOD) [11, 12].

### Data safety

To ensure proper handling of privacy-sensitive patient data, the independent Scientific Research Advisory Committee (Adviescommissie Wetenschappelijk Onderzoek—ACWO) within the OLVG was consulted and agreed (study number WO 21.099 – PURE) with the use of these data from the hospital population.

### Data source

A retrospective cohort study design was used, and data of 7.570 unique patients with documentation present in the database (Clarity) of the Electronical Medical Records (EMR) (EPIC, Wisconsin, United States) were extracted using a SQL query. Patients with a clinical admission at the department of Urology of a community hospital in Amsterdam between January 2015 and October 2021 were included. Patients that deceased during clinical admission were excluded. To prevent repeated measures and data leakage, one admission or readmission per patient was included in the dataset.

### Unplanned readmission

The primary outcome was a 30 day unplanned hospital readmission at the department of Urology, and readmissions were defined as clinical admissions within 30 days of discharge from previous clinical admission at the department of Urology.

### Features

Based on findings of several studies and clinical impact, 53 features were included, and some features, such as vitals or laboratory (lab) results, contained over time data within each admission.

These features are split into the following six categories:

- Patient characteristics
- Lab results
- Medication
- Health care logistics
- Medical history
- Type of surgery

(For a detailed overview, see Appendix.)

### Bias

Possible bias could originate from arbitrarily choosing a set of features by the researchers, incomplete documentation of data on features, and unknown lab results from external parties that were not included.

### Missing data

Missing data, was checked for the Missing At Random (MAR) assumption, and platelet count (82.6% missing) was dropped as feature. All remaining continuous features with missing data (serum creatinine, hemoglobin, BMI, alcohol use, systolic and diastolic blood pressure, and smoking history), were imputed using multiple imputation by chained equations [13] (MICE) with a default number of multiple imputations (5), 100 iterations (maxit), and the Predictive Mean Matching (PMM) settings for imputing numerical data. Non-continuous features with missing data were coded to ‘No’ or ‘Absent’, and therefore showed no missing data. More information considering imputed features can be found in Table 1.

**Table 1:** Missing values per feature: count and percentage

*Variable*	*Count*	*Percentage*
Platelet count	6254	82.6
Serum creatinine	4050	53.5
Hemoglobin	3742	49.4
BMI	2187	28.9
Alcohol use	1645	21.7
Systolic blood pressure	902	11.9
Diastolic blood pressure	902	11.9
Smoking history	507	6.7

### Study size

Specific information about patient characteristics can be found in Table 3 in Appendix.

### Imbalanced outcome

Of all observations, 10% of all patients had an unplanned readmission. This indicates a class imbalance and poses a potential problem when performing classification, as classification leans towards the class with the most observations and can skew the performance of an algorithm [14]. Observations on outcome were rebalanced using Synthetic Minority Oversampling Technique (SMOTE) and synthetized observations (i.e. oversampling) based on existing observations, combined with removing existing observations (i.e. undersampling) to create a specified balance. To prevent data leakage, data was split into a train and test set and resampling was only performed on the training set. Patients with an unplanned readmission were oversampled to 36% and patients without an unplanned readmission were set to 64% using undersampling.

### Model development

For modelling and evaluating, only supervised ML was applied. To achieve the first aim of this study, developing a PURE probability calculator, the following regression algorithms were used: 1) Logistic Regression, Penalized Logistic Regression 2) LASSO, and 3) RIDGE. The following classification algorithms were used: 4) Normal Decision Tree, 5) Bagged Trees, 6) Boosted Trees, 7) XG Boosted Trees, and 8) Random Forest. The available data was split to a ratio of 70:30 to create a training, and test set respectively. More information concerning patient characteristics between the train- and the test data can be found in the Appendix in Table 4. To ensure a fitting sampling strategy, 5-fold cross validation on the training set was applied. Before using the data for training and evaluating the models, all data were corrected for outliers and examined for confounding using correlation analysis and Principal Component Analysis (PCA) [15]. Centering and scaling was configured as extra setting in the regression algorithms to apply during training. Feature engineering (variable selection) was evaluated using the RandomForest algorithm to identify the predictive value for each feature, with importance measured in mean decrease of accuracy per feature [16].

### Model evaluation

To achieve the second aim of this study, evaluate differences in diagnostic performance characteristics of the regression and classification algorithms, the following metrics were used: accuracy, sensitivity, specificity, Area Under the Curve (AUC), Positive Predictive Value (PPV), and Negative Predictive Value (NPV).

### Software

Data pre-processing and analysis were performed using R Version 4.0.2, and R-studio Version 1.3.1073 (R-Studio, Boston, MA, USA). All code is made available via https://github.com/koenwelvaars/PURE_study.

## Results

In total, 7.570 unique patients were included with 52 different features.

### Study size

**Fig. 1:**
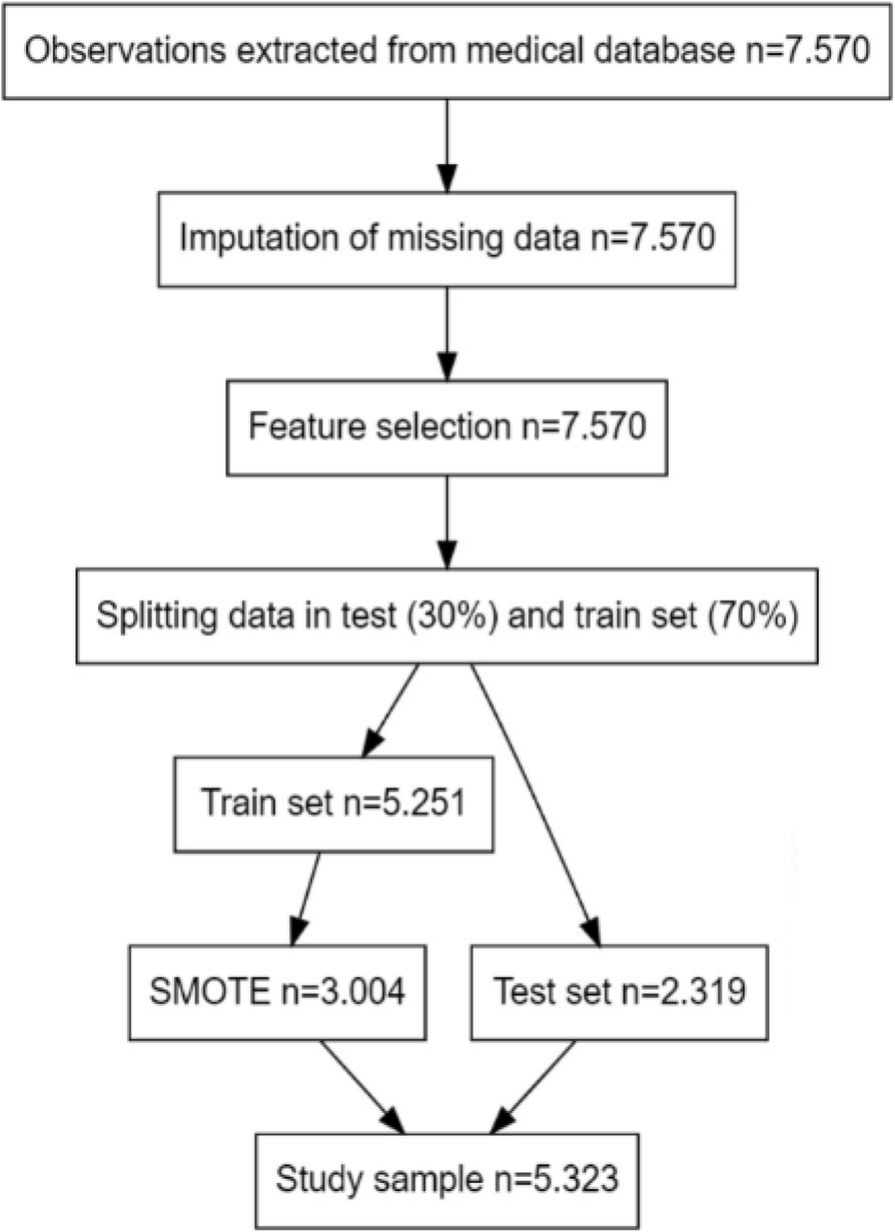
Flow diagram observations

Starting with 7.570 observations, the process of over and undersampling using SMOTE changed the original number observations. SMOTE was only applied to the train set to prevent leakage of information into the test set. In the training of models, 5.323 observations were included. More information on selection of observations and each taken step in this process is shown in Fig. 1.

### Feature selection

The feature importance of the 52 features were evaluated with a RandomForest algorithm training 2500 trees and features were included based on two criteria:

1. the feature had a good predictive value (> = 10% importance);
2. the feature was expected to have clinical importance.

**Fig. 2:**
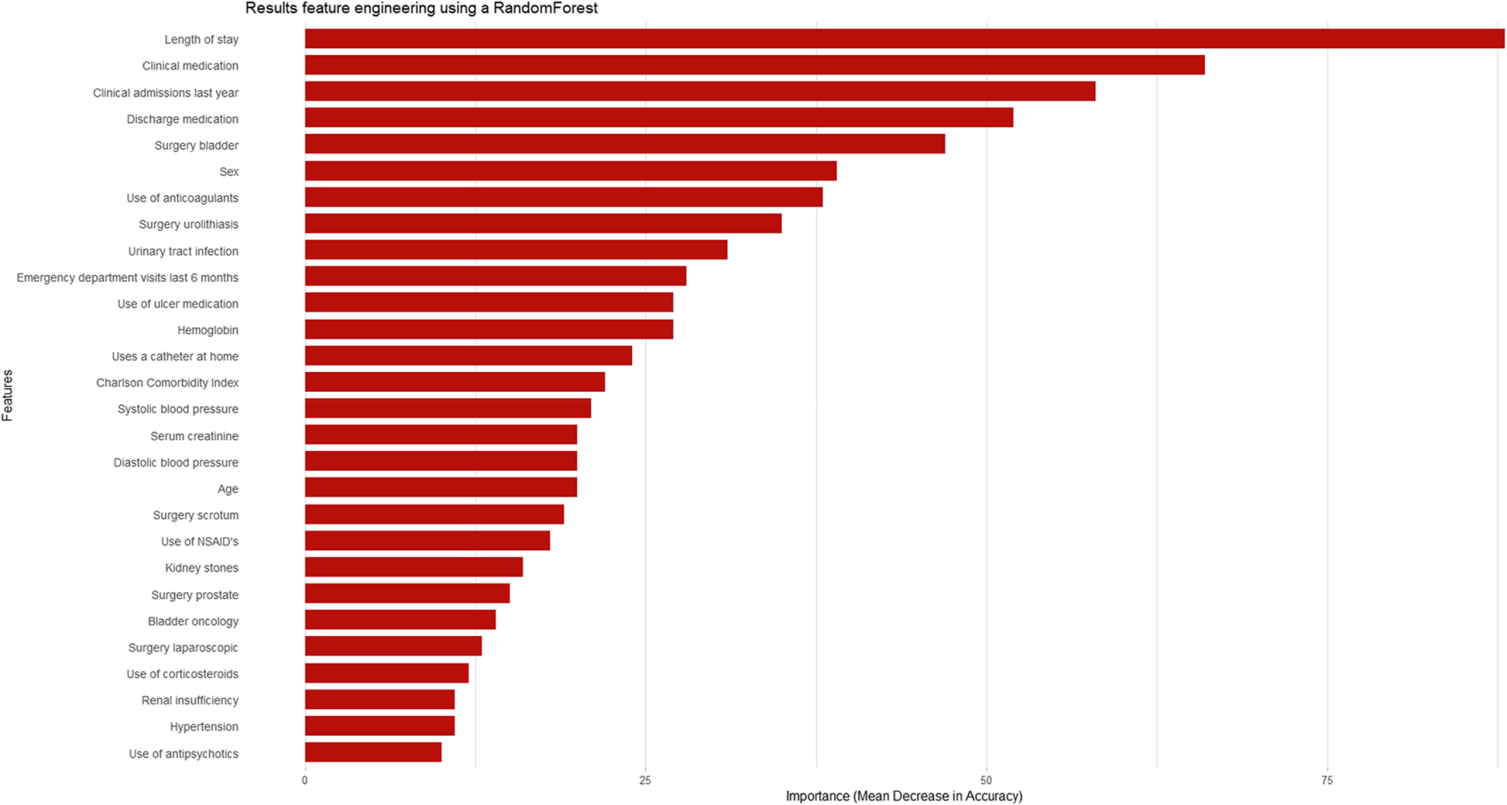
Feature importance

In the final model, 28 features were included ranging from length of stay to use of antipsychotics. Feature importance was calculated and the importance per feature can be found in Fig. 2. This figure indicates an overall performance per feature and does not indicate a negative or positive effect on outcome. Consult Fig. 5 in the Appendix for information on all features, where red features were included and blue features were not.

### Evaluate performance differences between regression and classification algorithms

To assess the baseline performance, models were trained on selected features and without hyperparameter tweaking. The only non-default setting was the number of trees (default is 500) as trained by the RandomForest algorithm, which was set to 2000.

**Fig. 3:**
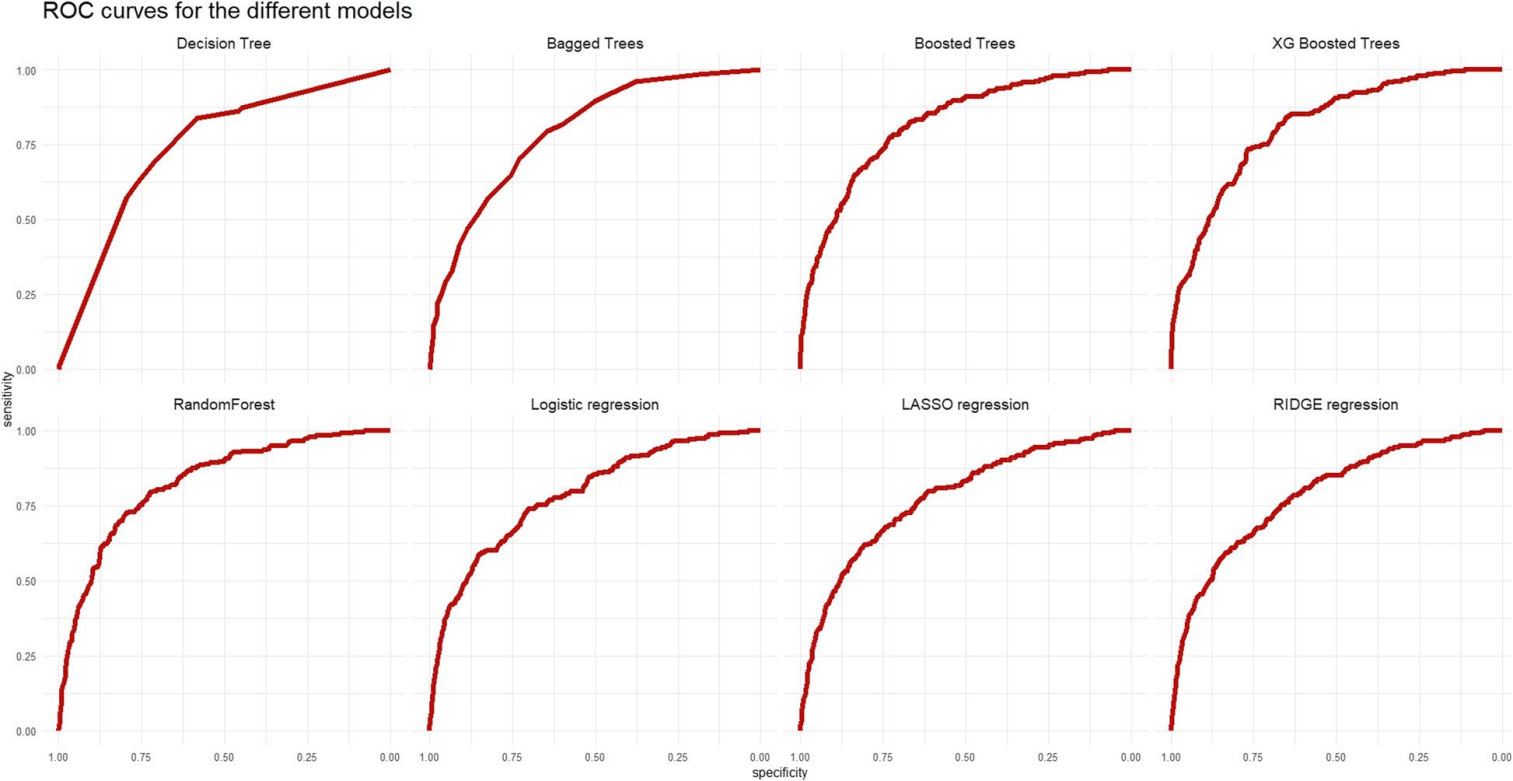
ROC curves of models

Evaluated on the test set, most models had good AUC scores ranging from 0.62 to 0.82. For AUC, a score above 0.80 indicates a strong discriminative ability. The models showed a better performance in predicting positives in comparison to negatives based on the balance between sensitivity and specificity. The Positive Predictive Value (PPV) scores for all models did not drop below 0.92, indicating that 92% of patients predicted positive were truly readmitted to the hospital. Information of other metrics are shown in Table 2. As seen in the ROC curve plot in Fig. 3, models trained based on classification algorithms (straight lines) show a stronger performance and outperform models trained on regression algorithms (dotted lines). A Wilcoxon test was used to test for statistically significant difference between metrics of the classification algorithms as a group (Decision tree, bagged trees, boosted trees, XG boosted trees, RandomForest), and regression algorithms as a group (Logistic regression, LASSO, and RIDGE regression). Only specificity showed a statistically significant difference with a *p*-value of 0.0358, whereas sensitivity, AUC, PPV, and NPV did not (*p*-values of 0.1314, 0.0512, 0.1745, 0.0583, 0.0714 respectively).

**Table 2:** Evaluation of performance of regression and classification algorithms

Algorithm	Accuracy	Sensitivity	Specificity	AUC	PPV	NPV
Decision Tree	0.68	0.80	0.57	0.75	0.94	0.24
Bagged Trees	0.70	0.83	0.57	0.79	0.94	0.27
Boosted Trees	0.74	0.85	0.63	0.82	0.95	0.31
XG Boosted Trees	0.72	0.84	0.60	0.81	0.95	0.30
RandomForest	0.72	0.88	0.55	0.71	0.95	0.34
Logistic regression	0.71	0.88	0.53	0.79	0.94	0.32
LASSO regression	0.62	0.96	0.28	0.62	0.92	0.45
RIDGE regression	0.62	0.97	0.27	0.62	0.92	0.50

**Fig. 4:**
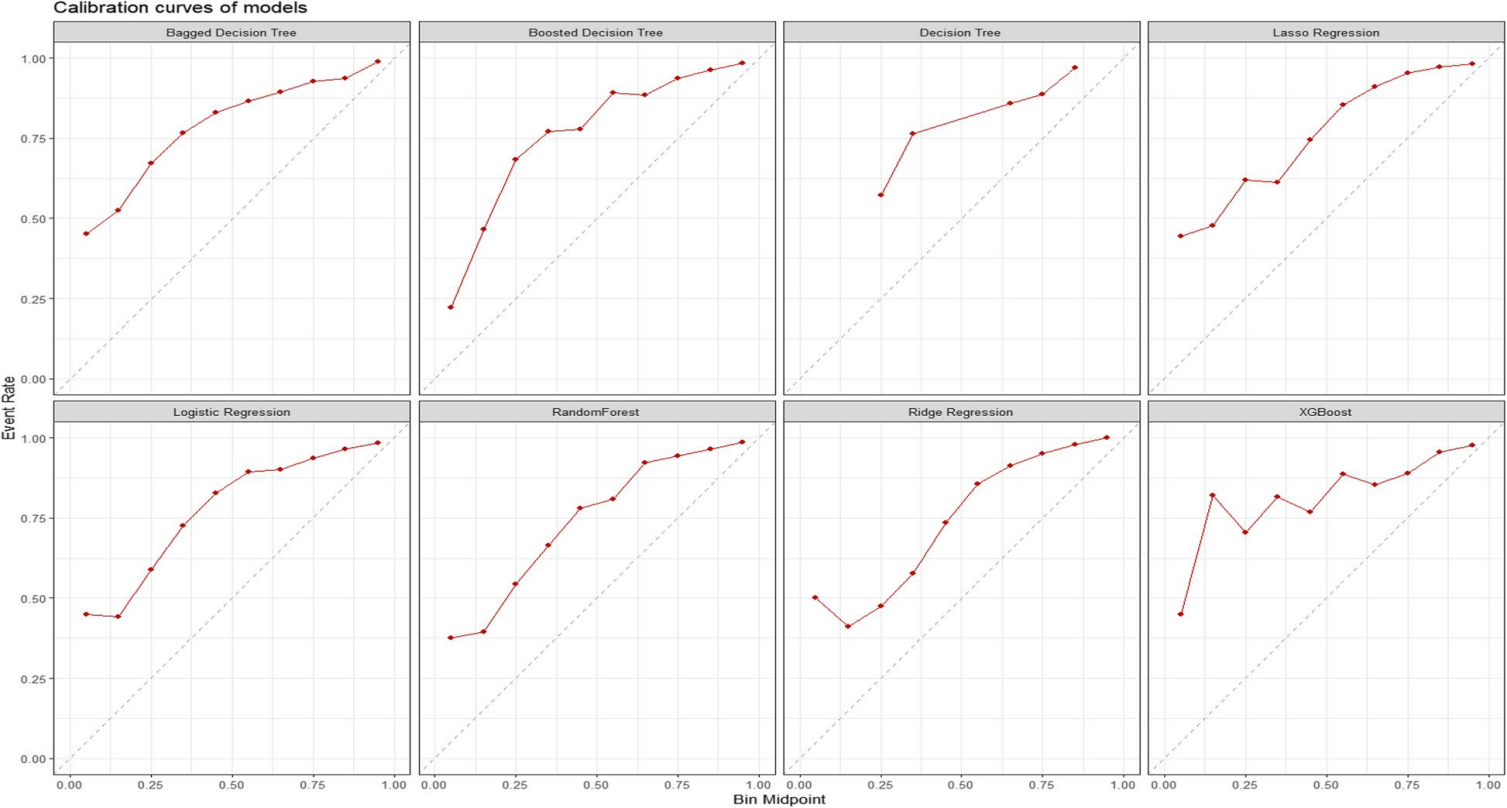
Calibration curves of models

**Fig. 5:**
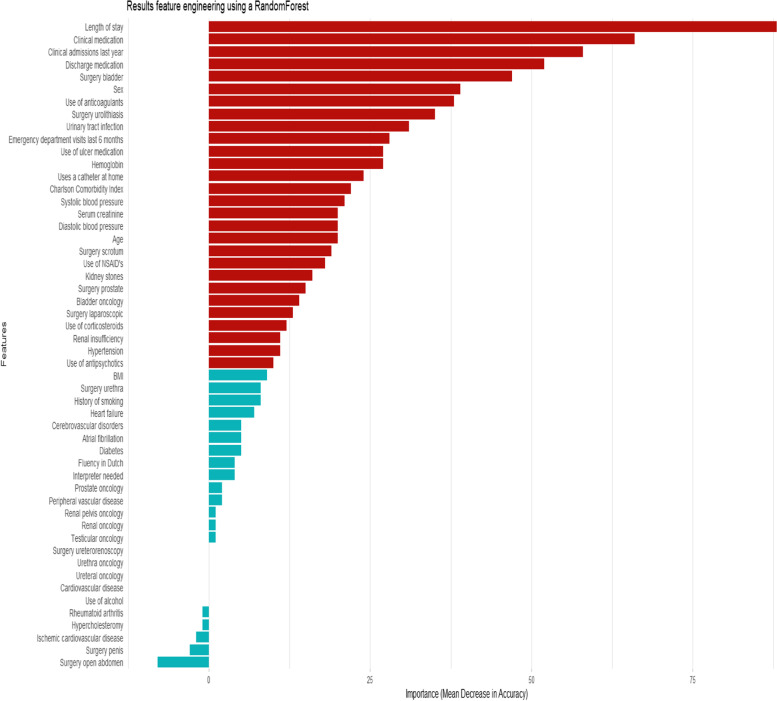
Importance of all features

The calibration curves of all trained models show that resampling with SMOTE mainly created an underestimation of predicting positives for our case of 30-day unplanned readmissions. If left without additional calibration, this would lead to a scenario where there would be few patients with a prediction of high risk of having a 30-day unplanned readmission. More information can be found in Fig. 4.

### Evaluation of the final model used as probability calculator for unplanned readmissions withing 30 days

An XGBoost model, a serial tree-based ensemble learner, showed the strongest overall performance and was chosen as the final model. The model using a boosted trees algorithm also shows a strong performance, but was not chosen due to three reasons being 1) less robust to overfitting, 2) cannot apply cross validation on each iteration, and 3) performs less accurate as compared to XGBoost on smaller datasets.

To assess whether performance of the XGBoost model can be improved, an automated grid search was executed on the train set to tune hyperparameters. The final model with optimized hyperparameters was evaluated on the test set and resulted in an improvement of 11% on accuracy (0.83) while other metrics showed similar performances, indicating that the original XGBoost model already had a strong overall performance. Additional information of the hyperparameters can be found in the Appendix. To assess performance bias in the final model, additional subgroup analysis were performed on sex, age groups, and surgery (yes/no). Statistical differences between the original dataset and subgroups were measured using DeLong’s test to compare two ROC curves. Within the subgroup sex, both male and female showed no significant difference with *p*-values of 0.4084 and 0.1428 respectively. Age was categorized into groups 18 – 45, 45 – 65, and 65 + , and showed no significant differences with *p*-values 0.0951, 0.8226, and 0.3019 respectively. Participants with surgery were compared to participants with no surgery and with *p*-values of 0.8182, and 0.5023 no significant differences were found. No subgroup analysis was performed on COVID-19 since inclusion of patients was limited to the department of Urology and did not suffer in patient care as compared to the department of Pulmonary Diseases for example.

## Discussion

Predictive models based on classification algorithms have a stronger performance compared to regression algorithms. The best performing model, the XGBoost model, had good diagnostic performance characteristics that can safely be applied as a risk calculator in clinical setting.

For the clinical department of Urology, evidence on applied ML in predicting unplanned readmissions is scarce. This is the first ML driven probability calculator with accurate prediction of unplanned readmission for Urology patients. Our study shows similar results (AUC 0.62 – 0.82) as compared to earlier studies on performance of predicting 30-day unplanned readmissions (AUC 0.21 – 0.88) [1]. Also, results on features having a high importance on outcome (e.g. length of stay, previous admission and medication) were comparable. We found that using a broader set of features led to a stronger performance as compared to only using LACE, and provides a more detailed risk stratification [9].

### Limitations

The results of this study should be interpreted in light of strengths and weaknesses. Strengths being an elaborate comparison using a multitude of features and ML techniques to develop models with. Weaknesses being the quality and presence of patient data on features, and no implementation of PURE in clinical practice to investigate transitioning from predicting to preventing unplanned readmissions.

Features with high importance do not show causal relationship and do not compare to features investigation in a randomized controlled trial. Therefore, feature importance should be evaluated thoroughly on model performance and clinical utility. The selection of features was partly arbitrarily chosen based on earlier scientific findings, and if expected to have a relevant clinical impact based on experiences from the clinical staff of Urology. Missing values of non-continuous features were coded to ‘No’ or ‘Absent’, and could show an incorrect importance as a consequence of incomplete discrete documentation of data in the patient chart. Based on clinical experience and discharge management in the hospital, a period was applied to extract mean values of the last 24 h before discharge in order to make use of features with over time data (e.g. blood pressure). This poses a problem for generalizing our findings, since other hospitals could apply a different period and a set of discharge management choices.

Most ML applications are specific and opt to improve patient care concerning patients suffering from urolithiasis, renal cell carcinoma, bladder cancer, and prostate cancer. As a more generic problem, prevention of unplanned readmissions by applying ML should be further studied in order to evaluate the efficacy on functional outcomes, reduce avoidable stress for patients and improve patient satisfaction [17]. In addition, shared decision making using risk-stratifying predictions of a ML model can decrease the risk up to 13%. Physicians are able to optimize specific outcomes (e.g. complications, infections) more easily by using a calculated risk stratification individual patient level, and discuss these findings with the patient in order to create awareness of potential risks [7, 18–20].

Aside from developing a best performing model, more investigation is necessary in order to determine what features lead to an improved performance. Also, the positive or negative impact of features on outcome need to be elucidated for a better understanding of the clinical value. Follow up studies should focus on varying such dependencies with a more in depth analysis of feature selection, and evaluate if a similar performance as compared to the PURE model is still achieved. In order to transition from predicting to preventing unplanned readmissions, this in depth analysis should also include a comparison of impact of non-modifiable (i.e. static, cannot act on) *versus* modifiable (i.e. dynamic, can act on) features on model performance and clinical utility.

In order to assess generalizability of the findings in our study, external validation by deploying the model using the same parameter settings and features, is a step that needs to be taken using a specific data sampling method. Other studies show similarities in improved results by applying resampling, but not much drift in calibration, suggesting that the impact of resampling effects on calibration are more case-sensitive as compared to other evaluation metrics. Although distorting calibration, our models trained on resampled data can still have clinical utility whereas the model can have poor calibration yet a strong discriminating performance [21, 22]. Hospitals have differences in patient population, discharge management, and even clinical workflows, which could affect performance of the model. Using transfer learning (i.e. the application of knowledge gained from completing one task to help solve a related problem), our model can be deployed in other hospitals and should be compared an evaluated if the same performance is acquired.

### Overall conclusion

It is feasible to develop a risk calculator with a strong performance in predicting unplanned readmissions for the department of Urology. In addition, regression based models are outperformed by classification based models and the latter should be a first pick for use of ML in order to predict unplanned readmissions.

## Data Availability

The data that support the findings of this study are available from OLVG but restrictions apply to the availability of these data, which were used under license for the current study, and so are not publicly available. Data are however available from the authors upon reasonable request and with permission of the ACWO. The code is accessible via https://github.com/koenwelvaars/PURE_study.

## Appendix

**Table 3:** Patient characteristics

	**Unplanned readmission within 30 days**			
	**Yes (** ***N*** ** = 774)**	**No (** ***N*** ** = 6796)**	***P*** **-value**	**Total (** ***N*** ** = 7570)**
**Charlson Comorbidity Index**				
Mean (SD)	1.48 (2.03)	0.998 (1.74)	< 0.001	1.05 (1.77)
Median [Min, Max]	0 [0, 10.0]	0 [0, 11.0]		0 [0, 11.0]
**Age**				
Mean (SD)	70.3 (15.7)	64.4 (17.5)	< 0.001	65.0 (17.4)
Median [Min, Max]	74.0 [20.0, 103]	68.0 [13.0, 109]		69.0 [13.0, 109]
**BMI**				
Mean (SD)	26.4 (5.48)	25.9 (4.92)	0.0181	26.0 (4.99)
Median [Min, Max]	25.6 [13.3, 66.5]	25.3 [13.3, 53.1]		25.3 [13.3, 66.5]
**Systolic blood pressure**				
Mean (SD)	134 (17.2)	131 (18.8)	< 0.001	131 (18.7)
Median [Min, Max]	133 [93.0, 182]	129 [85.0, 210]		129 [85.0, 210]
**Diastolic blood pressure**				
Mean (SD)	74.5 (8.18)	74.4 (9.04)	0.644	74.4 (8.96)
Median [Min, Max]	74.0 [53.0, 105]	74.0 [44.0, 126]		74.0 [44.0, 126]
**Creatinine blood**				
Mean (SD)	115 (90.9)	95.5 (64.2)	< 0.001	97.5 (67.7)
Median [Min, Max]	91.0 [37.0, 1260]	83.0 [21.0, 1480]		84.0 [21.0, 1480]
**Hemoglobin**				
Mean (SD)	7.67 (1.12)	7.72 (1.22)	0.246	7.71 (1.21)
Median [Min, Max]	7.70 [4.10, 11.6]	7.80 [4.00, 11.6]		7.80 [4.00, 11.6]
**Clinical medication**				
Mean (SD)	51.7 (34.0)	30.4 (25.7)	< 0.001	32.6 (27.4)
Median [Min, Max]	44.0 [7.00, 227]	22.0 [0, 267]		24.0 [0, 267]
**Home medication**				
Mean (SD)	12.8 (8.47)	8.07 (7.33)	< 0.001	8.55 (7.59)
Median [Min, Max]	11.0 [0, 48.0]	6.00 [0, 60.0]		6.00 [0, 60.0]
**Clinical admissions last year**				
Mean (SD)	0.860 (1.52)	0.311 (0.732)	< 0.001	0.367 (0.862)
Median [Min, Max]	0 [0, 11.0]	0 [0, 9.00]		0 [0, 11.0]
**ED visits last 6 months**				
Mean (SD)	0.382 (0.890)	0.144 (0.503)	< 0.001	0.169 (0.560)
Median [Min, Max]	0 [0, 8.00]	0 [0, 8.00]		0 [0, 8.00]
**Length of Stay**				
Mean (SD)	3.98 (5.44)	2.21 (3.35)	< 0.001	2.39 (3.66)
Median [Min, Max]	3.00 [0, 97.0]	1.00 [0, 65.0]		1.00 [0, 97.0]
**Sex**				
Female	153 (19.8%)	2439 (35.9%)	< 0.001	2592 (34.2%)
Male	621 (80.2%)	4357 (64.1%)		4978 (65.8%)
**History of smoking**				
No	656 (84.8%)	5577 (82.1%)	0.0702	6233 (82.3%)
Yes	118 (15.2%)	1219 (17.9%)		1337 (17.7%)
**Use of alcohol**				
No	420 (54.3%)	3308 (48.7%)	0.00363	3728 (49.2%)
Yes	354 (45.7%)	3488 (51.3%)		3842 (50.8%)
**Interpreter needed**				
No	738 (95.3%)	6618 (97.4%)	0.00183	7356 (97.2%)
Yes	36 (4.7%)	178 (2.6%)		214 (2.8%)
**Fluency in Dutch**				
No	78 (10.1%)	783 (11.5%)	0.255	861 (11.4%)
Yes	696 (89.9%)	6013 (88.5%)		6709 (88.6%)
**Uses a catheter at home**				
No	716 (92.5%)	6563 (96.6%)	< 0.001	7279 (96.2%)
Yes	58 (7.5%)	233 (3.4%)		291 (3.8%)
**Use of anticoagulants**				
No	116 (15.0%)	2392 (35.2%)	< 0.001	2508 (33.1%)
Yes	658 (85.0%)	4404 (64.8%)		5062 (66.9%)
**Use of NSAID's**				
No	529 (68.3%)	4697 (69.1%)	0.692	5226 (69.0%)
Yes	245 (31.7%)	2099 (30.9%)		2344 (31.0%)
**Use of corticosteroids**				
No	686 (88.6%)	6533 (96.1%)	< 0.001	7219 (95.4%)
Yes	88 (11.4%)	263 (3.9%)		351 (4.6%)
**Use of antipsychotics**				
No	715 (92.4%)	6578 (96.8%)	< 0.001	7293 (96.3%)
Yes	59 (7.6%)	218 (3.2%)		277 (3.7%)
**Use of ulcer medication**				
No	380 (49.1%)	4086 (60.1%)	< 0.001	4466 (59.0%)
Yes	394 (50.9%)	2710 (39.9%)		3104 (41.0%)
**Oncology**				
Absent	700 (90.4%)	6358 (93.6%)	0.0014	7058 (93.2%)
Present	74 (9.6%)	438 (6.4%)		512 (6.8%)
**Medication**				
No	83 (10.7%)	1793 (26.4%)	< 0.001	1876 (24.8%)
Yes	691 (89.3%)	5003 (73.6%)		5694 (75.2%)
**Comorbidity**				
Absent	607 (78.4%)	5995 (88.2%)	< 0.001	6602 (87.2%)
Present	167 (21.6%)	801 (11.8%)		968 (12.8%)
**Surgery**				
No	354 (45.7%)	4325 (63.6%)	< 0.001	4679 (61.8%)
Yes	420 (54.3%)	2471 (36.4%)		2891 (38.2%)

*P*-values calculated with Student’s T-test for numeric variables and Chi-squared test for categorical variables

Detailed overview of explanatory variables.

- Patient characteristics
  - ◦ Age
  - ◦ Sex
  - ◦ Charlson Comorbidity Index (CCI)
  - ◦ BMI
  - ◦ Smoking history
  - ◦ Use of alcohol
  - ◦ Fluency in Dutch
- Lab results during clinical admission
  - ◦ Mean diastolic blood pressure within 24 hours before discharge
  - ◦ Mean systolic blood pressure within 24 hours before discharge
  - ◦ Mean platelet count within 24 hours before discharge
  - ◦ Last serum creatinine before discharge
  - ◦ Last hemoglobin before discharge
- Currently active medication during admission
  - ◦ Total count of clinical medications
  - ◦ Total count of discharge medications
  - ◦ Use of anticoagulants
  - ◦ Use of NSAID’s
  - ◦ Use of corticosteroids
  - ◦ Use of antipsychotics
  - ◦ Use of ulcer medication
- Health care logistics at the time of admission
  - ◦ Total count of clinical admissions in the last year
  - ◦ Total count of emergency department visits last 6 months
  - ◦ Total length of stay
  - ◦ Interpreter needed
  - ◦ Home use of catheter
- Medical history
  - ◦ Hypercholesteremia
  - ◦ Diabetes type I or type II
  - ◦ Hypertension
  - ◦ Rheumatoid arthritis
  - ◦ Atrial fibrillation
  - ◦ Renal insufficiency
  - Cerebrovascular disorders
  - ◦ Ischemic cardiovascular disease
  - ◦ Peripheral vascular disease
  - ◦ Heart failure
  - ◦ Cardiovascular disease
  - ◦ Kidney stones
  - ◦ Urinary tract infection
  - ◦ Testicular oncology
  - ◦ Bladder oncology
  - ◦ Ureteral oncology
  - ◦ Urethra oncology
  - ◦ Renal oncology
  - ◦ Prostate oncology
  - ◦ Renal pelvis oncology
- Type of surgery
  - ◦ Open abdomen
  - ◦ Laparoscopic
  - ◦ Scrotum
  - ◦ Penis
  - ◦ Prostrate
  - ◦ Urethral
  - ◦ Ureterorenoscopy
  - ◦ Urolithiasis
  - ◦ Bladder

Patient characteristics between the train and the test dataset can be found in Table 4.

**Table 4:** Patient characteristics between train and test data

	**Train (*****N***** = 6008)**	**Test (*****N***** = 4638)**	***P*****-value**	**Total (*****N***** = 10,646)**
**Use of antipsychotics**				
No	5710 (95.0%)	4492 (96.9%)	< 0.001	10,202 (95.8%)
Yes	298 (5.0%)	146 (3.1%)		444 (4.2%)
**Hypertension**				
No	5718 (95.2%)	4420 (95.3%)	0.796	10,138 (95.2%)
Yes	290 (4.8%)	218 (4.7%)		508 (4.8%)
**Renal insufficiency**				
No	5830 (97.0%)	4514 (97.3%)	0.405	10,344 (97.2%)
Yes	178 (3.0%)	124 (2.7%)		302 (2.8%)
**Use of corticosteroids**				
No	5632 (93.7%)	4440 (95.7%)	< 0.001	10,072 (94.6%)
Yes	376 (6.3%)	198 (4.3%)		574 (5.4%)
**Surgery laparoscopic**				
No	5862 (97.6%)	4528 (97.6%)	0.896	10,390 (97.6%)
Yes	146 (2.4%)	110 (2.4%)		256 (2.4%)
**Bladder oncology**				
No	5734 (95.4%)	4464 (96.2%)	0.0441	10,198 (95.8%)
Yes	274 (4.6%)	174 (3.8%)		448 (4.2%)
**Surgery prostate**				
No	5318 (88.5%)	4220 (91.0%)	< 0.001	9538 (89.6%)
Yes	690 (11.5%)	418 (9.0%)		1108 (10.4%)
**Kidney stones**				
No	5860 (97.5%)	4484 (96.7%)	0.00982	10,344 (97.2%)
Yes	148 (2.5%)	154 (3.3%)		302 (2.8%)
**Use of NSAID's**				
No	4140 (68.9%)	3214 (69.3%)	0.682	7354 (69.1%)
Yes	1868 (31.1%)	1424 (30.7%)		3292 (30.9%)
**Surgery scrotal**				
No	5874 (97.8%)	4516 (97.4%)	0.203	10,390 (97.6%)
Yes	134 (2.2%)	122 (2.6%)		256 (2.4%)
**Age**				
Mean (SD)	66.7 (16.8)	65.0 (17.7)	< 0.001	65.9 (17.2)
Median [Min, Max]	71.0 [13.0, 103]	69.0 [17.0, 106]		70.0 [13.0, 106]
**Diastolic blood pressure**				
Mean (SD)	74.2 (8.85)	74.7 (8.89)	0.00368	74.5 (8.87)
Median [Min, Max]	74.0 [44.0, 126]	74.0 [44.0, 117]		74.0 [44.0, 126]
**Creatinine blood**				
Mean (SD)	104 (80.5)	97.7 (62.9)	< 0.001	101 (73.5)
Median [Min, Max]	87.0 [21.0, 1270]	84.0 [28.0, 1480]		85.0 [21.0, 1480]
**Systolic blood pressure**				
Mean (SD)	132 (18.2)	132 (18.7)	0.956	132 (18.4)
Median [Min, Max]	130 [87.0, 210]	130 [85.0, 200]		130 [85.0, 210]
**Charlson Comorbidity Index**				
Mean (SD)	1.15 (1.83)	1.04 (1.80)	0.00404	1.10 (1.82)
Median [Min, Max]	0 [0, 11.0]	0 [0, 10.0]		0 [0, 11.0]
**Uses a catheter at home**				
No	5758 (95.8%)	4432 (95.6%)	0.509	10,190 (95.7%)
Yes	250 (4.2%)	206 (4.4%)		456 (4.3%)
**Hemoglobin**				
Mean (SD)	7.71 (1.14)	7.69 (1.19)	0.629	7.70 (1.16)
Median [Min, Max]	7.80 [4.00, 11.6]	7.80 [4.00, 11.6]		7.80 [4.00, 11.6]
**Use of ulcer medication**				
No	3324 (55.3%)	2764 (59.6%)	< 0.001	6088 (57.2%)
Yes	2684 (44.7%)	1874 (40.4%)		4558 (42.8%)
**ED visits last 6 months**				
Mean (SD)	0.207 (0.594)	0.162 (0.558)	< 0.001	0.188 (0.579)
Median [Min, Max]	0 [0, 8.00]	0 [0, 7.00]		0 [0, 8.00]
**Urinary tract infection**				
No	5818 (96.8%)	4530 (97.7%)	0.0115	10,348 (97.2%)
Yes	190 (3.2%)	108 (2.3%)		298 (2.8%)
**Surgery urolithiasis**				
No	5462 (90.9%)	4232 (91.2%)	0.572	9694 (91.1%)
Yes	546 (9.1%)	406 (8.8%)		952 (8.9%)
**Use of anticoagulants**				
No	1550 (25.8%)	1608 (34.7%)	< 0.001	3158 (29.7%)
Yes	4458 (74.2%)	3030 (65.3%)		7488 (70.3%)
**Sex**				
Female	1832 (30.5%)	1556 (33.5%)	< 0.001	3388 (31.8%)
Male	4176 (69.5%)	3082 (66.5%)		7258 (68.2%)
**Surgery bladder**				
No	4744 (79.0%)	3878 (83.6%)	< 0.001	8622 (81.0%)
Yes	1264 (21.0%)	760 (16.4%)		2024 (19.0%)
**Home medication**				
Mean (SD)	9.83 (7.80)	8.55 (7.62)	< 0.001	9.27 (7.75)
Median [Min, Max]	8.00 [0, 51.0]	6.00 [0, 59.0]		7.00 [0, 59.0]
**Clinical admissions last year**				
Mean (SD)	0.479 (1.03)	0.361 (0.838)	< 0.001	0.428 (0.952)
Median [Min, Max]	0 [0, 11.0]	0 [0, 11.0]		0 [0, 11.0]
**Clinical medication**				
Mean (SD)	37.6 (29.8)	33.0 (27.9)	< 0.001	35.6 (29.1)
Median [Min, Max]	28.0 [0, 253]	25.0 [0, 209]		26.0 [0, 253]
**Length of Stay**				
Mean (SD)	2.84 (3.72)	2.39 (3.75)	< 0.001	2.64 (3.74)
Median [Min, Max]	2.00 [0, 65.0]	1.00 [0, 97.0]		2.00 [0, 97.0]
**Oncology**				
Absent	5734 (95.4%)	4464 (96.2%)	0.0441	10,198 (95.8%)
Present	274 (4.6%)	174 (3.8%)		448 (4.2%)
**Medication**				
No	1182 (19.7%)	1200 (25.9%)	< 0.001	2382 (22.4%)
Yes	4826 (80.3%)	3438 (74.1%)		8264 (77.6%)
**Comorbidity**				
Absent	5422 (90.2%)	4158 (89.7%)	0.326	9580 (90.0%)
Present	586 (9.8%)	480 (10.3%)		1066 (10.0%)
**Surgery**				
No	3588 (59.7%)	2988 (64.4%)	< 0.001	6576 (61.8%)
Yes	2420 (40.3%)	1650 (35.6%)		4070 (38.2%)

*P*-values calculated with Student’s T-test for numeric variables and Chi-squared test for categorical variables

Detailed informed of hyperparameter optimization of the XGBoost model.

A grid-search was performed on the train set using 5-fold CV, to search for optimal parameter settings. Optimal parameter values found were: nrounds = 3000, eta = 0.015, max_depth = 5, gamma = 0.05, colsample_bytree = 1, min_child_weight = 1, and subsample = 0.5.

Importance of all features is shown in Fig. 5. This step was performed before feature selection for developing the models. 

## Abbreviations

PURE: Predicting unplanned readmissions
ML: Machine learning
